# A novel artificial intelligence-based endoscopic ultrasonography diagnostic system for diagnosing the invasion depth of early gastric cancer

**DOI:** 10.1007/s00535-024-02102-1

**Published:** 2024-05-07

**Authors:** Ryotaro Uema, Yoshito Hayashi, Takashi Kizu, Takumi Igura, Hideharu Ogiyama, Takuya Yamada, Risato Takeda, Kengo Nagai, Takuya Inoue, Masashi Yamamoto, Shinjiro Yamaguchi, Takashi Kanesaka, Takeo Yoshihara, Minoru Kato, Shunsuke Yoshii, Yoshiki Tsujii, Shinichiro Shinzaki, Tetsuo Takehara

**Affiliations:** 1 Department of Gastroenterology and Hepatology, Osaka University Graduate School of Medicine, 2-2, Yamadaoka, Suita, Osaka 565-0871 Japan; 2 Department of Gastroenterology, Yao Municipal Hospital, Yao, 581-0069 Japan; 3 Department of Gastroenterology, Sumitomo Hospital, Osaka, 530-0005 Japan; 4 Department of Gastroenterology, Ikeda Municipal Hospital, Ikeda, 563-0025 Japan; 5 Department of Gastroenterology, Osaka Rosai Hospital, Sakai, 591-8025 Japan; 6 Department of Gastroenterology, Itami City Hospital, Itami, 664-0015 Japan; 7 Department of Gastroenterology, Suita Municipal Hospital, Suita, 564-0018 Japan; 8 Department of Gastroenterology, Osaka General Medical Center, Osaka, 558-8558 Japan; 9 Department of Gastroenterology, Toyonaka Municipal Hospital, Toyonaka, 560-8565 Japan; 10 Department of Gastroenterology, Kansai Rosai Hospital, Amagasaki, 660-0064 Japan; 11 Department of Gastrointestinal Oncology, Osaka International Cancer Institute, Osaka, 540-0008 Japan; 12 Department of Gastroenterology, Faculty of Medicine, Hyogo Medical University, Nishinomiya, 663-8501 Japan

**Keywords:** Endoscopic ultrasonography, Early gastric cancer, Artificial intelligence, Deep learning

## Abstract

**Background:**

We developed an artificial intelligence (AI)-based endoscopic ultrasonography (EUS) system for diagnosing the invasion depth of early gastric cancer (EGC), and we evaluated the performance of this system.

**Methods:**

A total of 8280 EUS images from 559 EGC cases were collected from 11 institutions. Within this dataset, 3451 images (285 cases) from one institution were used as a development dataset. The AI model consisted of segmentation and classification steps, followed by the CycleGAN method to bridge differences in EUS images captured by different equipment. AI model performance was evaluated using an internal validation dataset collected from the same institution as the development dataset (1726 images, 135 cases). External validation was conducted using images collected from the other 10 institutions (3103 images, 139 cases).

**Results:**

The area under the curve (AUC) of the AI model in the internal validation dataset was 0.870 (95% CI: 0.796–0.944). Regarding diagnostic performance, the accuracy/sensitivity/specificity values of the AI model, experts (*n* = 6), and nonexperts (*n* = 8) were 82.2/63.4/90.4%, 81.9/66.3/88.7%, and 68.3/60.9/71.5%, respectively. The AUC of the AI model in the external validation dataset was 0.815 (95% CI: 0.743–0.886). The accuracy/sensitivity/specificity values of the AI model (74.1/73.1/75.0%) and the real-time diagnoses of experts (75.5/79.1/72.2%) in the external validation dataset were comparable.

**Conclusions:**

Our AI model demonstrated a diagnostic performance equivalent to that of experts.

**Supplementary Information:**

The online version contains supplementary material available at 10.1007/s00535-024-02102-1.

## Introduction

Gastric cancer is a leading cause of death in the Asian region and ranks fifth in incidence and fourth in mortality worldwide [1]. The treatment strategy for early gastric cancer (EGC) patients is mainly determined by the depth of cancer invasion. Specifically, lymph node metastasis is infrequent in EGC with mucosal (M) or slight submucosal (SM) invasion (<500 μm from the muscularis mucosae); thus, endoscopic submucosal dissection (ESD) can be indicated as the treatment [2]. Therefore, evaluating the depth of invasion into the SM layer is crucial, and endoscopic ultrasonography (EUS) is widely used for depth assessment.

EUS is used for the T staging of EGC, as it provides information about the deeper layers of the gastric wall [3]. In particular, EUS using miniature probes is widely used to determine the invasion depth of EGC [4]. We have previously reported a combination strategy consisting of conventional endoscopy (CE) and EUS, in which EUS is performed only on patients with suspected deep SM invasion during CE [5], and we have also reported the clinical usefulness of EUS in a prospective study [6]. Although the diagnostic accuracy of EUS has been reported to be approximately 70–90% [5–12], some studies have shown a high accuracy of over 90% [13, 14], while others have reported a low accuracy of less than 70% [15, 16], indicating inconsistency. This is attributed to the fact that EUS depends heavily on the diagnostic skill of the physician. To obtain an accurate diagnosis by EUS, sufficient experience and knowledge of EUS images of gastric cancer are necessary [17]. To compensate for this extensive experience and knowledge, the development of computer-aided diagnostic systems for EUS has been desired.

In recent years, artificial intelligence (AI) utilizing deep learning [18] has made remarkable progress in the medical field. In the field of gastric cancer, there have been several reports of the use of AI for detecting lesions [19–21], differentiating between cancer and noncancer [22, 23], delineating lateral cancer margins [24, 25], and diagnosing the invasion depth [26–28]. However, there have been no reports on the application of AI in diagnostic EUS. The aim of this study was to develop an AI system for diagnosing EGC using EUS and to verify its effectiveness.

## Methods

### Study design and patients

We identified consecutive cases of EGC in which EUS was performed using a miniature probe at Osaka University between June 2009 and December 2019 to create a dataset for developing and validating the AI system. The exclusion criteria were as follows: (1) no endoscopic or surgical resection performed; (2) absence of evaluable images; (3) images from second or subsequent EUS examinations of the same lesion; (4) no evidence of cancer in the resected specimen; and (5) difficulty determining corresponding lesions in cases of multiple lesions.

As an external validation cohort, we used EUS images from EGC patients prospectively enrolled between May 2017 and January 2021 at 11 institutions from our previous study (UMIN000025862) [6]. In that study, EGC patients with suspected SM invasion on screening endoscopy were enrolled, and the exclusion criteria were as follows: (1) previous gastrectomy or esophagectomy, (2) suspected local recurrence, (3) suspected special histological type of EGC, such as neuroendocrine carcinoma, GC with lymphoid stroma, or GC of fundic gland type, (4) no expected treatment within 8 weeks of diagnosis, and (5) serious complications or multiple active cancers for whom EGC treatment is impractical. Among the enrolled patients, those who met the following criteria were excluded from the present study: (1) examination performed at Osaka University; (2) no endoscopic or surgical resection performed; and (3) inability to collect EUS images. We excluded cases from Osaka University because some of them were included in the development and internal validation datasets. EUS images of all eligible cases were retrospectively collected and used for external validation.

This study was approved by the ethics committee of Osaka University (No. 20324 and No. 22028) and performed in accordance with the Declaration of Helsinki guidelines. The requirement of informed consent was waived for this study, and all participants were given the opportunity to refuse participation using an opt-out method on the website of each institute.

### EUS procedure and diagnosis

Following the diagnostic procedure by CE, EUS was performed using miniature probes with a frequency of 20 MHz or 12 MHz (UM-2R, frequency 12 MHz, UM-3R, frequency 20 MHz, or UM-DP20-25R, frequency 20 MHz: Olympus Corporation; P-2226-12, frequency 12 MHz or P-2226-20, frequency 20 MHz: Fujifilm Corporation) and an ultrasound system (EU-M2000 or EU-ME1 or EU-ME2: Olympus Corporation; SP-702 or SP-900: Fujifilm Corporation). In principle, the examination was ordinarily performed with a 20 MHz probe; only when detailed observation was difficult, it was performed with a 12 MHz probe. Lesions with the third layer of the five separated layers showing invagination, thinning, or complete destruction were diagnosed as SM2 (SM2; ≥500 μm SM invasion from the muscularis mucosae) or deeper. Otherwise, lesions were diagnosed as M-SM1 (SM1; <500 μm SM invasion from the muscularis mucosae) because the differentiation between M and SM1 is difficult with EUS. As a result, all lesions were classified as “M-SM1” or “SM2 or deeper.”

### Construction of the dataset

The images collected at Osaka University were divided by period and used as the development and internal validation datasets. We excluded images that depicted lesions other than the target lesion, noisy or blurred images, and images with annotations such as arrows and text. We used all remaining images, including images that appeared to have captured normal mucosa around the target lesion and low-quality images that were inappropriate for diagnosis.

**Fig. 1:**
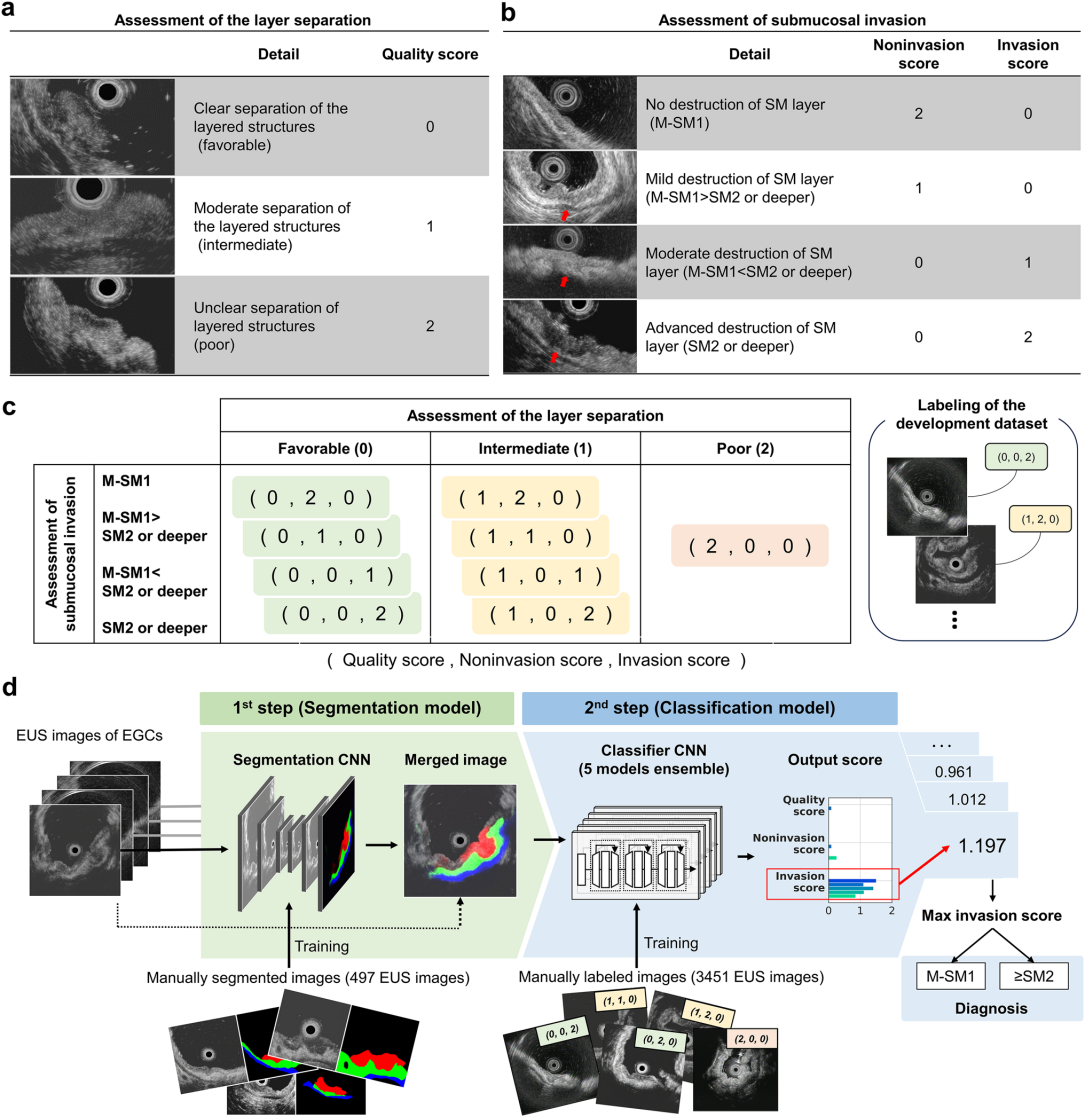
An overview of the labeling of the dataset and the AI model used in this study. **a** Scoring of layer separation (quality score), which was classified into three categories: favorable (0), intermediate (1), and poor (2). **b** Scoring of submucosal invasion (noninvasion and invasion scores), which was categorized into four groups based on the degree of destruction of the submucosal layer. If the quality score was 2, it was difficult to evaluate invasion, and both the noninvasion and invasion scores were set to 0. **c** All combinations of the scores used in this study. All EUS images in the development dataset were labeled with one of these tags. **d** Overview of the AI system developed in this study. The EUS images were first input into the segmentation model (1st step), which segmented the tumor, submucosal layer, and muscular layer. The output images were merged with the original images and then input into the classification model (2nd step), which provided the quality score, noninvasion score, and invasion score for each image. The scores were output for all images of each lesion, and the highest invasion score determined whether the lesion was classified as “M-SM1” or “SM2 or deeper”. M-SM1, mucosal cancer or cancer in the submucosa <500 μm from the muscularis mucosae; SM2, cancer in the submucosa ≥500 μm from the muscularis mucosae; EUS, endoscopic ultrasonography; EGC, early gastric cancer; CNN, convolutional neural network

Subsequently, all EUS images in the development dataset were scored by an expert gastroenterologist based on the histological invasion depth. Due to substantial variability in EUS images regarding the suspicion of invasion and their suitability for diagnosis, it is not feasible to assess them with a simple binary value of presence or absence of invasion. Therefore, we utilized a three-vector scoring system as described below: quality score (the quality of visualization, such as layer separation), noninvasion score (the possibility of no SM invasion), and invasion score (the possibility of SM invasion) (Fig. 1a, b). Specifically, the quality was scored as 0 (favorable), 1 (intermediate), or 2 (poor) based on the quality of layer separation (Fig. 1a). The possibility of SM invasion was evaluated based on the degree of destruction of the submucosal layer as follows: no destruction of the submucosal layer and no suspicion of invasion, M-SM1 (noninvasion score: 2, invasion score: 0); slight destruction with possible invasion, M-SM1 > SM2 or deeper (noninvasion score: 1, invasion score: 0); moderate destruction with suspected invasion, M-SM1 < SM2 or deeper (noninvasion score: 0, invasion score: 1); and severe destruction with obvious invasion, SM2 or deeper (noninvasion score: 0, invasion score: 2) (Fig. 1b). However, in images where the quality of layer separation was poor (quality score: 2), it was difficult to evaluate invasion; therefore, the scores were both set to 0 (noninvasion score: 0, invasion score: 0). All combinations of scores used in this study are shown in Fig. 1c. For some of the images in the development dataset, we manually segmented the tumor, submucosal layer, and muscular layer to train the segmentation model. For the internal and external validation datasets, we merely labeled the depth information of the lesions without performing image-level labeling or segmentation.

### Development of the AI system

We utilized PyTorch (https://pytorch.org/), a deep learning framework, to develop the AI system. In this study, we constructed the AI system as a two-step diagnostic system using convolutional neural networks (Fig. 1d). The first step consisted of a segmentation model that mapped the tumor, submucosal layer, and muscular layer in EUS images. The network of the segmentation model used UNET with ResNet34 as the backbone. The input image was resized to a square of 512 × 512 pixels, and we trained the model to maximize the Dice coefficient using the Adam optimizer. To prevent overfitting, we trained the model with data augmentation techniques such as HorizontalFlip, ShiftScaleRotate, and RandomBrightnessContrast. The map images output from UNET were mixed with the original EUS images at a ratio of 1.0:0.2 and then used as input for the following step. The parameters of the training procedure are given in Supplementary Table 1. The output images from the first step were resized to 224 × 224 pixels and then input into the second step.

The second step consisted of a classification model that simultaneously output the quality score (0–2), noninvasion score (0–2), and invasion score (0–2). The network of the classification model used a pretrained EfficientNetV2-L model. We removed the original fully connected layer and added a new fully connected layer that contained a hidden layer of 128 nodes. For parameter tuning, we split the development dataset into 5 groups and performed fivefold cross-validation. All original layers of EfficientNetV2-L and the new fully connected layer were trained. We trained the model to maximize the mean AUC of the three scores using the rectified Adam (RAdam) optimizer and root mean square error as the loss function. To prevent overfitting, we trained the model with data augmentation techniques such as HorizontalFlip, ShiftScaleRotate, and RandomBrightnessContrast. The parameters of the training procedure are given in Supplementary Table 2. Finally, we used an ensemble model that consisted of 5 models obtained from the fivefold cross-validation as our AI model. Averaging was used as the ensemble technique. Based on the data exploration in the development dataset, the maximum invasion score for each lesion was found to particularly contribute to the depth of invasion (see Supplementary Method; Supplementary Fig. 1). Therefore, only the invasion score was used for depth of invasion diagnosis, and the other two scores were not utilized. All training and inference were performed in a local environment using an Intel Core i9-12900K as the central processing unit and a GeForce RTX3090 as the graphics processing unit.

### Visualization of regions of interest (ROIs) for the AI model using class activation mapping (CAM)

To investigate the ROI of the developed AI model, we performed visualization using CAM. In this study, we employed the Eigen-CAM method of CAM. We obtained the feature maps corresponding to the output of each class and weighted the output value by multiplying it by the class output. We obtained these maps for each of the 5 models in the ensemble model and averaged them to create a visualization map for the input image. We implemented these codes using the PyTorch-grad-cam library for PyTorch (https://github.com/jacobgil/pytorch-grad-cam).

### Training of CycleGAN model

We addressed the domain shift problem of the external validation dataset by using CycleGAN [29]. We used all EUS images derived from the EU-M2000 system (Olympus) in the development and internal validation datasets as well as all EUS images derived from the EU-ME1 and EU-ME2 systems (Olympus) in the external validation dataset as the training dataset for CycleGAN. We trained the model for a total of 30 epochs, with each epoch consisting of the full set of images. We implemented these codes using PyTorch-CycleGAN-and-pix2pix (https://github.com/junyanz/pytorch-CycleGAN-and-pix2pix).

### Outcome measures

The primary outcome was the diagnostic performance of the developed AI system for the classification of “M-SM1” and “SM2 or deeper” per lesion. As a secondary outcome, we compared the diagnostic performance of the AI system with that of gastroenterologists. In the internal validation dataset, we compared the diagnostic abilities of the AI system, six expert gastroenterologists, and eight nonexpert gastroenterologists. The expert gastroenterologists were those who met all of the following criteria: (1) more than 10 years of experience in gastrointestinal endoscopy, (2) experience with more than 30 cases of EUS for EGC, and (3) board certification as a fellow of the Japan Gastroenterological Endoscopy Society. Nonexpert gastroenterologists were those who did not meet at least one of these requirements. For internal validation, both expert and nonexpert gastroenterologists reviewed only all EUS images of each lesion and classified each lesion as either “M-SM1” or “SM2 or deeper.” When the diagnosis differed between images, the diagnosis was based on the image that appeared to reflect the deepest area of the lesion. For external validation, real-time EUS diagnoses by expert gastroenterologists at each institution were used.

In the AI system, an inference process was performed for all images of each lesion using the developed model, and the maximum invasion score was considered the score for that lesion. The diagnosis of “M-SM1” or “SM2 or deeper” was determined based on whether the score exceeded a threshold value. We calculated the diagnostic performance for all values of the invasion score and adopted the point closest to the performance of the experts as the threshold value.

### Statistical analysis

We compared the performance of the AI system and gastroenterologists by calculating the accuracy, sensitivity, specificity, positive predictive value (PPV), and negative predictive value (NPV). The 95% confidence intervals (CIs) of those indicators were also calculated. Pearson’s chi-square test and the McNemar test were used to compare the diagnostic performance among evaluators. The receiver operating characteristic (ROC) curve and area under the curve (AUC) were used to represent the classification performance of our model using Python. A *p* value less than 0.05 was considered statistically significant. All statistical analyses were performed using JMP Pro version 16 (SAS Institute, Inc., Cary, NC, USA) and R version 4.2.1 (The R Foundation for Statistical Computing, Vienna, Austria).

## Results

### Patient and lesion characteristics

**Fig. 2:**
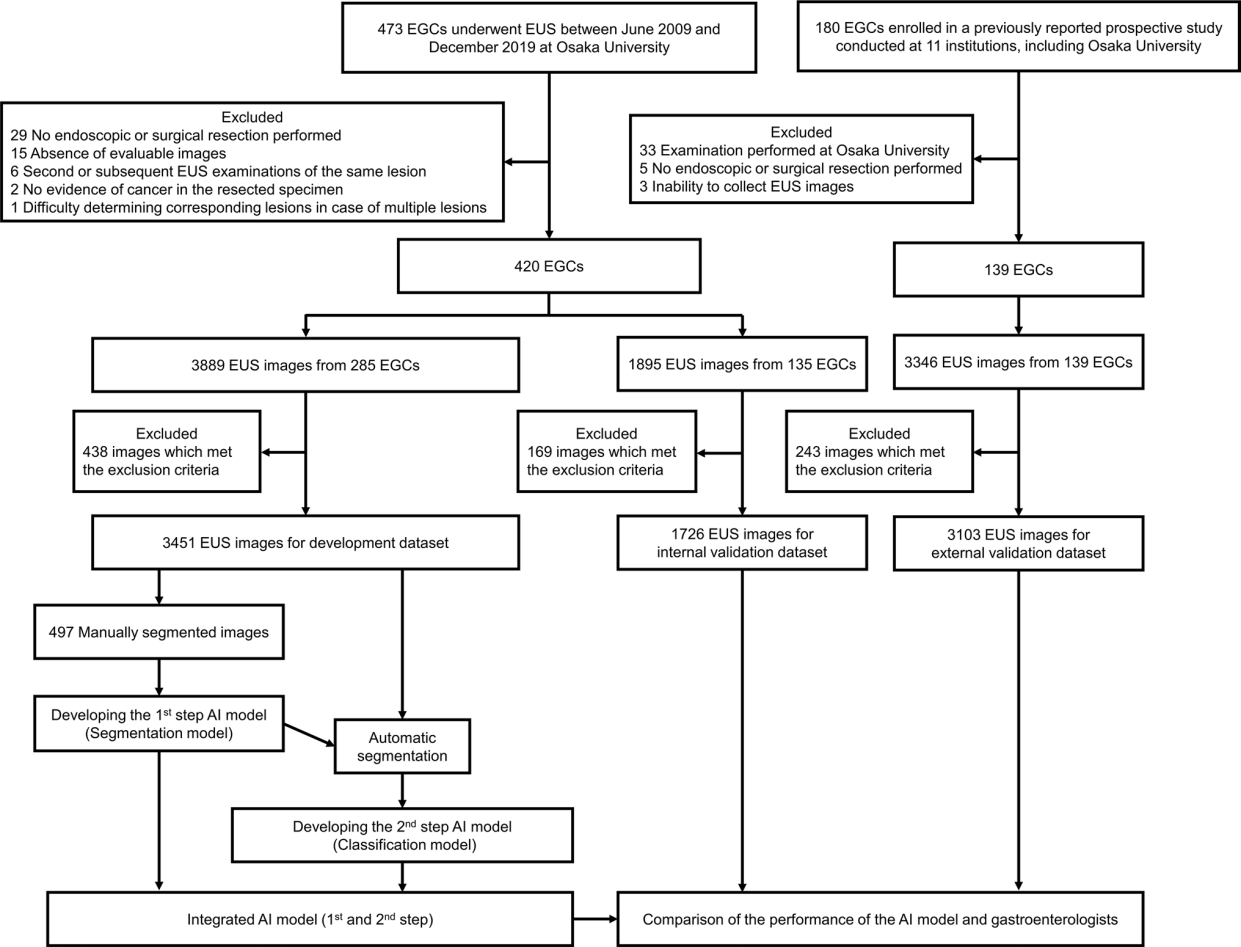
Patient flowchart of this study. EUS, endoscopic ultrasonography; EGC, early gastric cancer

Figure 2 shows the patient flowchart. A total of 285 patients with 3451 images were obtained as the development dataset, and all images were scored using the aforementioned criteria. Among these, manual mapping of the mucosal layer, submucosal layer, and muscular layer in 497 images was performed for segmentation model training. A total of 180 patients with 1726 images were obtained as the internal validation dataset. Regarding the external validation dataset, among all 180 patients enrolled in the previously reported prospective study, we used 3103 EUS images from 139 patients. Of the 9130 collected EUS images, 851 (9.3%) images met the exclusion criteria and were excluded. The clinical characteristics of the development dataset, the internal validation dataset, and the external validation dataset are presented in Supplementary Table 3. The proportions of lesions with SM2 or deeper in the training, internal validation, and external validation datasets were 24%, 28%, and 48%, respectively.

### Internal validation

**Fig. 3:**
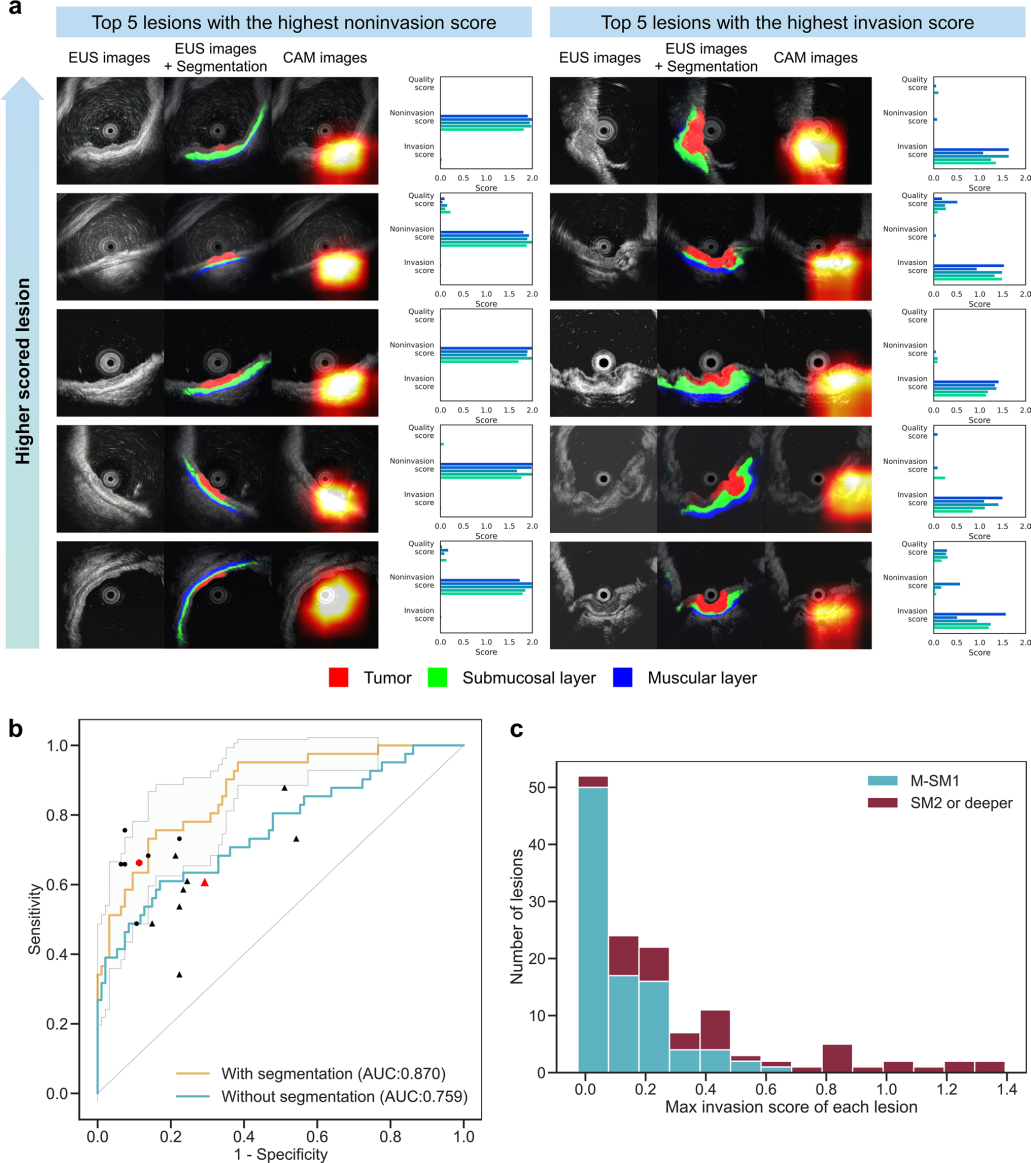
Examples of output images from the AI model and diagnostic performance of the AI model and gastroenterologists in the internal validation dataset. **a** Top five lesions based on the noninvasion score (all lesions were histologically M-SM1) and top five lesions based on the invasion score (all lesions were histologically SM2 or deeper). For each image, the input EUS image, the segmentation map inferred by the segmentation model, the ROI visualized by CAM, and the output score are presented. These examples demonstrate that the AI model accurately recognized the layer structure and destruction of the submucosal layer. **b** ROC curve of the AI model for diagnosing “M-SM1” and “SM2 or deeper.” The light blue area enclosed by dotted lines indicates the 95% confidence interval for the AI model with segmentation model. The AI model achieved an AUC of 0.870. Without applying the segmentation model, the AUC decreased to 0.759. The diagnostic performance of experts and nonexperts is represented by circles and triangles, respectively, with the red shape indicating the mean value for each group. **c** Histogram of the highest invasion score for each lesion plotted separately for “M-SM1” and “SM2 or deeper”. The proportion of lesions with SM2 or deeper invasion increased as the invasion score increased. M-SM1, mucosal cancer or cancer in the submucosa <500 μm from the muscularis mucosae; SM2, cancer in the submucosa ≥500 μm from the muscularis mucosae; EUS, endoscopic ultrasonography; ROI, region of interest; CAM, class activation mapping; ROC, receiver operating characteristic; AUC, area under the curve

We present examples of the output data of our AI model for the internal validation dataset in Fig. 3a. We computed the maximum noninvasion score and invasion score for each lesion and showed the top five lesion images for each. The AI model appropriately segmented the layered structure and recognized sites where SM invasion was suspected. We also presented an example of our model in the Supplementary Video.

We applied the AI model to all images in the internal validation dataset (*n* = 135). The diagnostic performance of the AI model was sufficient, with an AUC of 0.870 (95% CI: 0.796–0.944) (Fig. 3b). We also evaluated the diagnostic performance of a model trained solely on raw images without segmented images. The model trained with segmented images showed a significantly higher AUC than the model trained without them (0.870 vs. 0.759, *p* < 0.001, DeLong test). Furthermore, the AI model outperformed all nonexperts and demonstrated diagnostic performance equivalent to that of experts (Fig. 3b). The proportion of patients with SM2 or deeper invasion increased with an increasing invasion score (Fig. 3c). When the invasion score threshold was set to 0.3, the accuracy, sensitivity, and specificity of the AI system, experts, and nonexperts were 82.2/63.4/90.4%, 81.9/66.3/88.7%, and 68.3/60.9/71.5%, respectively (Table 1). The AI model showed significantly higher accuracy than nonexperts, while no significant difference was observed between the AI model and experts. The diagnostic performance for all values of the invasion score is shown in Supplementary Table 4.

**Table 1:** Diagnostic performance of the developed AI model and gastroenterologists on the internal validation dataset

	Diagnostic performance, % (95% CI)					
	Accuracy	*p* value^1^	Sensitivity	Specificity	PPV	NPV
Developed AI model	82.2 (74.7–88.3)	Reference	63.4 (46.9–77.9)	90.4 (82.6–95.5)	74.3 (56.7–87.5)	85.0 (76.5–91.4)
Average of experts (*n* = 6)	81.9	0.95	66.3	88.7	72.8	85.7
Average of nonexperts (*n* = 8)	68.3	<0.01	60.9	71.5	50.0	81.3

*AI* artificial intelligence, *PPV* positive predictive value, *NPV* negative predictive value

^1^ The *p* value was calculated using Pearson’s chi-square test in comparison with the accuracy of the developed AI model

### External validation and domain adaptation with CycleGAN

**Fig. 4:**
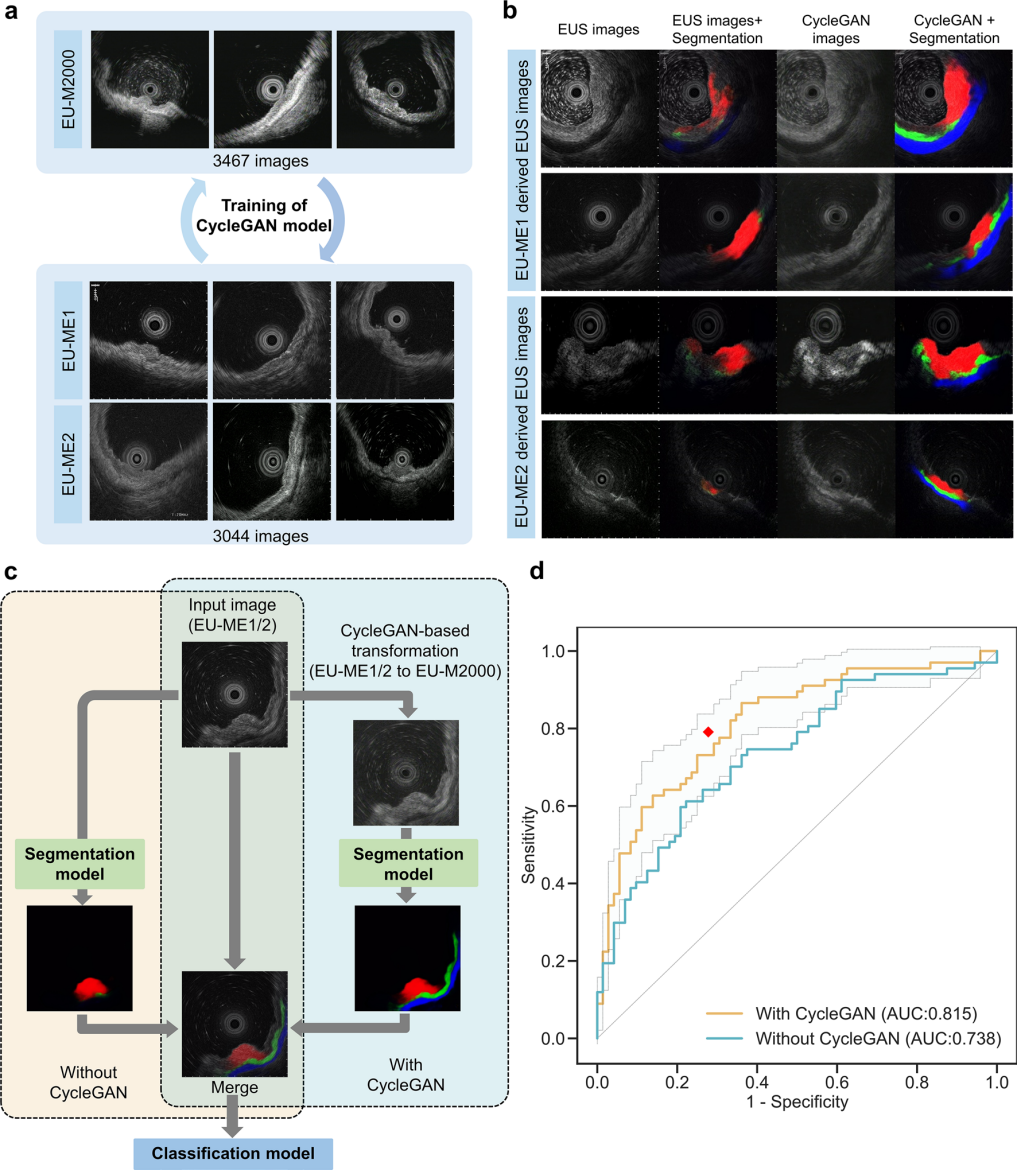
Domain adaptation of the external validation dataset using CycleGAN and diagnostic performance of the AI model and experts in the external validation dataset. **a** All EU-ME1/ME2-derived EUS images from the external dataset and all EU-M2000-derived EUS images from the internal dataset were used as the training data for CycleGAN model. **b** Comparison of segmentation maps when applying the segmentation model to raw EUS images and CycleGAN-transformed EUS images. The recognition accuracy was significantly improved after CycleGAN transformation. **c** The input EUS images were merged with the segmentation images with/without CycleGAN-based transformation and input into the classification model. **d** ROC curve of the AI model for diagnosing “M-SM1” and “SM2 or deeper”. The light blue area enclosed by dotted lines indicates the 95% confidence interval for the AI model with CycleGAN. The AUC was 0.738 when CycleGAN was not applied, but it increased to 0.818 after applying CycleGAN. The red dots represent the EUS-based diagnostic performance of the experts from each facility at the time of case registration during the prospective study. ROC, receiver operating characteristic; AUC, area under the curve; M-SM1, mucosal cancer or cancer in the submucosa <500 μm from the muscularis mucosae; SM2, cancer in the submucosa ≥500 μm from the muscularis mucosae

We validated the performance of the AI model in the external validation dataset. Details of the EUS images collected from each institute, such as EUS equipment, number of lesions, and image size, are shown in Supplementary Table 5. Initially, we applied the AI model directly to the external validation dataset, but the AUC was insufficient, at 0.738 (95% CI: 0.655–0.821). One possible factor was the difference in EUS equipment used between the internal and external validation datasets (Supplementary Table 3). In the external validation dataset, most images were obtained with the EU-ME1 and EU-ME2 systems, which were not used in the development dataset, and the segmentation quality was low for these images (Supplementary Fig. 2). To address this issue, we used a style transfer method based on a generative adversarial network (GAN) called CycleGAN [29] to convert images obtained using the EU-ME1 and EU-ME2 systems to the images obtained using the EU-M2000 system, which accounted for the majority of the development dataset (Fig. 4a). After application of the CycleGAN style transfer method, the quality of segmentation was improved (Fig. 4b; Supplementary Fig. 3). Subsequently, we evaluated the diagnostic performance of the AI model using composite images, which combined the original EUS images and the segmented images generated from the CycleGAN-based transformed images (Fig. 4c). As a result, the AUC of the AI model significantly increased to 0.815 (95% CI: 0.743–0.886) (0.815 vs. 0.738, *p* = 0.003, DeLong test) (Fig. 4d). With a cutoff value of 0.3 as in the internal validation dataset, the accuracy, sensitivity, and specificity of the AI model were 74.1%, 73.1%, and 75.0%, respectively, with no significant difference from the diagnosis of experts (*p* = 0.88) (Table 2).

**Table 2:** Diagnostic performance of the developed AI model and the real-time EUS diagnoses by experts on the external validation dataset

Evaluator	Diagnostic performance (95% CI)					
	Accuracy	*p* value^1^	Sensitivity	Specificity	PPV	NPV
Developed AI model	74.1 (66.0–81.2)	Reference	73.1 (60.9–83.2)	75.0 (63.4–84.5)	73.1 (60.9–83.2)	75.0 (63.4–84.5)
Experts (10 institutions)	75.5 (67.5–82.4)	0.88	79.1 (67.4–88.1)	72.2 (60.4–82.1)	72.6 (60.9–82.4)	78.8 (67.0–87.9)

*AI* artificial intelligence, *PPV* positive predictive value, *NPV* negative predictive value

^1^ The *p* value was calculated using the McNemar test

### Diagnostic performance of the AI model and combination diagnostic algorithm

**Fig. 5:**
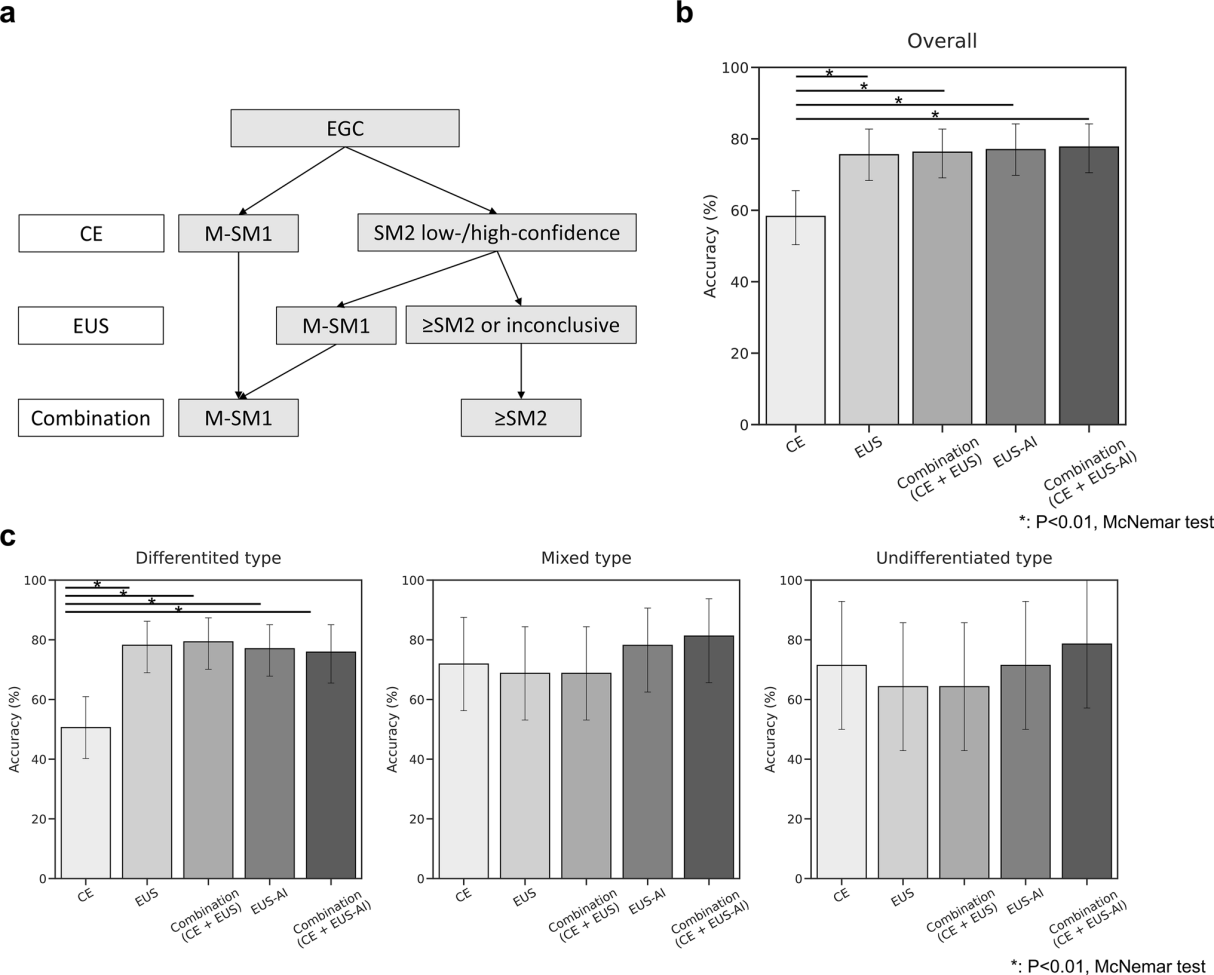
Diagnostic performance of a combined strategy using CE and EUS in the external validation dataset. **a** Integrated diagnostic algorithm combining CE and EUS. EUS was performed only for lesions with suspected SM invasion based on CE and for diagnosing SM2 or deeper invasion if also suspected on EUS. **b** Comparison of the diagnostic accuracy between gastroenterologists and the AI model for all lesions when the combined strategy of CE and EUS was applied. Experts’ real-time diagnoses at the time of case enrollment in the prospective study were used. The combined strategy showed a significantly improved accuracy compared to that of CE alone, and there was no significant difference between the experts and the AI model. **c** Comparison of the diagnostic performance of the combined strategy among different histological types. The combined strategy was effective for the differentiated type, but no additional benefit of EUS was observed for the mixed and undifferentiated types, which was consistent with a previous report. M-SM1, mucosal cancer or cancer in the submucosa <500 μm from the muscularis mucosae; SM2, cancer in the submucosa ≥500 μm from the muscularis mucosae; CE, conventional endoscopy; EUS, endoscopic ultrasonography

We previously proposed a diagnostic algorithm that combines CE and EUS and showed its usefulness (Fig. 5a). We then investigated whether incorporating the AI model into the combined algorithm would similarly result in improved diagnostic performance in the external validation cohort. The diagnostic accuracy using CE alone was 58.3%, whereas this value increased to 76.3% when combined with expert EUS-based diagnosis and 77.7% when combined with the AI model for diagnosis by EUS. In both cases, the diagnostic accuracy was significantly better than that of CE alone (58.3% vs. 76.3%, *p* < 0.001; 58.3% vs. 77.7%, *p* = 0.002) (Fig. 5b). We also evaluated the diagnostic accuracy by histological type (Fig. 5c). In the differentiated type, the diagnostic accuracy using CE alone was 50.6%, whereas it significantly increased to 72.4% when combined with the AI model (50.6% vs. 72.4%, *p* = 0.002), which was consistent with previously reported results [6]. In the mixed and undifferentiated types, there was no additional effect of combining CE with the AI model, consistent with a previous report [6].

## Discussion

In this study, we developed an AI-based EUS diagnostic system for staging the invasion depth of EGC. EUS diagnosis of early gastric cancer is not a simple task; however, by using an AI model with a segmentation step and innovating our labeling process, we demonstrated a diagnostic accuracy comparable to that of experts. Furthermore, we showed that our results were consistent with those of experts by using not only an internal validation dataset but also an external validation dataset. To our knowledge, this is the first report of an AI-based EUS diagnostic system for the T staging of EGC.

EUS for the diagnosis of EGC is crucial, especially in terms of assessing the invasion depth. However, much experience is required to obtain appropriate skills for staging by EUS [30]. In particular, proper identification of the layered structure of the gastric wall and the lesion is required to assess the invasion depth of EGC. Furthermore, the presence of ulcerative findings, which are characteristic of gastric cancer, can also complicate the diagnosis [11]. Not all gastroenterologists have sufficient opportunity to accumulate experience in their clinical practice. Therefore, an AI system that can assist in the diagnosis of EGC by EUS would be extremely useful for filling the gap in experience.

One of the greatest challenges in constructing an EUS-based diagnostic system is the labeling of training data. This is because EUS images often include images of both the lesion and the surrounding normal tissue. Furthermore, in many cases, the areas with cancer invasion account for only a small part of the entire EGC lesion; thus, it is also necessary to evaluate whether EUS images accurately visualize these areas of cancer invasion. Additionally, there are lesions for which most images are of poor quality and unsuitable for use in diagnosis. To overcome these difficulties, we developed a three-vector scoring system including the quality score, noninvasion score, and invasion score. The three scores exhibited a relationship of mutual exclusion, wherein a high quality score (indicating unsuitability for diagnosis) would result in low noninvasion and invasion scores (Supplementary Fig. 1b). As a result, only images truly capable of contributing to the diagnosis could be evaluated. In principle, labeling should be performed based on pathological depth as the gold standard. However, for the aforementioned reasons, it was difficult to simply associate each image with the corresponding pathological depth. Therefore, it was not possible to avoid subjective labeling by gastroenterologists. In the evaluation of the validation dataset, only the diagnostic accuracy for the depth of invasion of each lesion was assessed, and the importance of the validity of image-level labeling in this study was considered relatively low. One approach to overcome these issues is a method called multiple-instance learning (MIL) [31], which is often adapted to tasks such as whole-slide imaging. This method involves training the AI model by collecting multiple images into a single set and labeling them at the set level. Instead of labeling at the image level, it is possible to label the entire set of images for a case, allowing labeling to be performed without being influenced by the evaluator’s subjectivity. However, MIL often requires many datasets, and as a strategy in the context of limited training data, as in this study, the current method was considered optimal.

A distinctive feature of this study is the use of a two-step system consisting of a segmentation model and a classification model. Feeding segmented images into a subsequent image classifier is a common approach in the field of image recognition [32]. In the interpretation of EUS images, proper recognition of the wall structure is crucial. Therefore, we created an independent model to recognize the layered structure of the gastric wall. This approach improved the diagnostic accuracy (Fig. 3b). Identifying layers first and then evaluating the invasion depth is the same process that gastroenterologists use to diagnose patients based on EUS images, and this approach replicates their process of thinking.

In this study, the diagnostic performance of the AI model was improved by applying CycleGAN to the external validation dataset. In the field of machine learning, the decrease in performance that occurs when a trained AI model is applied to another dataset is called the domain shift problem [33]. We used a GAN-based technique to overcome this problem. GANs are AI models where two networks, the generator and the discriminator, compete to create and identify realistic outputs, respectively. CycleGAN, a variant of GAN, enables the conversion of images from one style to another without matched pairs using a parameter of cycle consistency, which checks that a converted image can be converted back to its original form [29]. In recent years, GAN-based techniques, including CycleGAN, have been reported as a way to overcome this domain shift problem [34], including in gastrointestinal endoscopy [35]. In this study, the AI model developed at Osaka University did not exhibit sufficient performance in the external validation cohort in its original state. The largest factor was considered the difference in the EUS equipment used between the institutions, and good results were achieved by learning the domain transformation of images obtained using different EUS equipment. In principle, it is ideal to address this issue by training the model with images obtained from all kinds of EUS equipment, but the images that can be obtained are often limited in the medical field. Thus, a method such as CycleGAN can be considered one potential approach.

Because EUS is a time-consuming procedure, it is important to perform it only in appropriate cases. As previously mentioned, we advocate for performing EUS only for patients with suspected deep SM invasion based on CE findings [5, 6]. In this study, we have shown that incorporating the developed AI system into this strategy could achieve diagnostic accuracy equivalent to that of experts (Fig. 5b). The external validation dataset used in this study was limited to patients with suspected deep SM invasion based on CE and did not include cases with obvious mucosal cancer. This was a very challenging condition for the AI model, but it is noteworthy that it achieved diagnostic accuracy equivalent to that of experts even with such a realistic dataset. Furthermore, in recent years, there have been multiple reports on the use of AI to diagnose the invasion depth of gastric cancer using CE [26–28]. Additionally, there have been reports on AI in video analysis [36] and the beneficial collaboration between AI and endoscopists [37]. Gong et al. [38] reported a real-time diagnostic accuracy of 86.4% in a large-scale prospective randomized trial. Thus, invasion depth diagnosis with AI for gastric cancer using CE has made important advancements. On the other hand, EUS plays a complementary role by providing information that CE cannot obtain. In the future, a collaboration between AI for CE and AI for EUS is expected to achieve higher diagnostic performance.

This study had several limitations. First, the development dataset was collected from a single institution. The quality of EUS images may vary among different institutions and equipment, but those variations were not fully reflected in our training data. However, we could overcome this limitation. Second, although our AI system demonstrated sufficient diagnostic performance, the amount of training data was relatively small compared to that used for general deep learning tasks. However, notably, the AI model achieved a diagnostic accuracy equivalent to that of experts using only approximately 3400 images. Third, this study was retrospective and used still images. Thus, whether this system is useful in actual real-time diagnosis is unknown, and further prospective studies are necessary. Fourth, we were unable to demonstrate the usefulness of the system for undifferentiated cancer. It has been reported that the diagnostic accuracy of EUS for undifferentiated cancer is not sufficient [39], which is considered a limitation of the EUS method. However, it is possible that the AI system with an increase in training data could overcome this issue, depending on further research.

In conclusion, our AI-based EUS diagnostic system for diagnosing the invasion depth of EGC demonstrated diagnostic performance equivalent to that of experts. This system may improve diagnostic accuracy when assessing the depth of invasion in EGC.

## Supplementary Information

Below is the link to the electronic supplementary material.

Supplementary file1 (DOCX 3764 KB)

Supplementary file2 (MP4 115259 KB)

## References

[1] Sung H, Ferlay J, Siegel RL, et al. Global cancer statistics 2020: GLOBOCAN estimates of incidence and mortality worldwide for 36 cancers in 185 countries. CA Cancer J Clin. 2021;71:209–49. 33538338 10.3322/caac.21660

[2] Japanese Gastric Cancer Association. Japanese gastric cancer treatment guidelines 2021 (6th edition). Gastric Cancer. 2023;26:1–25. 36342574 10.1007/s10120-022-01331-8 PMC9813208

[3] Cardoso R, Coburn N, Seevaratnam R, et al. A systematic review and meta-analysis of the utility of EUS for preoperative staging for gastric cancer. Gastric Cancer. 2012;15(Suppl 1):S19–26. 22237654 10.1007/s10120-011-0115-4

[4] Yanai H, Fujimura H, Suzumi M, et al. Delineation of the gastric muscularis mucosae and assessment of depth of invasion of early gastric cancer using a 20-megahertz endoscopic ultrasound probe. Gastrointest Endosc. 1993;39:505–12. 8365597 10.1016/s0016-5107(93)70160-1

[5] Tsujii Y, Kato M, Inoue T, et al. Integrated diagnostic strategy for the invasion depth of early gastric cancer by conventional endoscopy and EUS. Gastrointest Endosc. 2015;82:452–9. 25841580 10.1016/j.gie.2015.01.022

[6] Tsujii Y, Hayashi Y, Ishihara R, et al. Diagnostic value of endoscopic ultrasonography for the depth of gastric cancer suspected of submucosal invasion: a multicenter prospective study. Surg Endosc. 2022;37:3018–28. 36536083 10.1007/s00464-022-09778-7

[7] Yanai H, Noguchi T, Mizumachi S, et al. A blind comparison of the effectiveness of endoscopic ultrasonography and endoscopy in staging early gastric cancer. Gut. 1999;44:361–5. 10026321 10.1136/gut.44.3.361 PMC1727404

[8] Matsumoto Y, Yanai H, Tokiyama H, et al. Endoscopic ultrasonography for diagnosis of submucosal invasion in early gastric cancer. J Gastroenterol. 2000;35:326–31. 10832666 10.1007/s005350050356

[9] Hizawa K, Iwai K, Esaki M, et al. Is endoscopic ultrasonography indispensable in assessing the appropriateness of endoscopic resection for gastric cancer? Endoscopy. 2002;34:973–8. 12471541 10.1055/s-2002-35851

[10] Kim JH, Song KS, Youn YH, et al. Clinicopathologic factors influence accurate endosonographic assessment for early gastric cancer. Gastrointest Endosc. 2007;66:901–8. 17963876 10.1016/j.gie.2007.06.012

[11] Okada K, Fujisaki J, Kasuga A, et al. Endoscopic ultrasonography is valuable for identifying early gastric cancers meeting expanded-indication criteria for endoscopic submucosal dissection. Surg Endosc. 2011;25:841–8. 20734082 10.1007/s00464-010-1279-4

[12] Kim J, Kim SG, Chung H, et al. Clinical efficacy of endoscopic ultrasonography for decision of treatment strategy of gastric cancer. Surg Endosc. 2018;32:3789–97. 29435750 10.1007/s00464-018-6104-5

[13] Yoshida S, Tanaka S, Kunihiro K, et al. Diagnostic ability of high-frequency ultrasound probe sonography in staging early gastric cancer, especially for submucosal invasion. Abdom Imaging. 2005;30:518–23. 15688103 10.1007/s00261-004-0287-z

[14] Mouri R, Yoshida S, Tanaka S, et al. Usefulness of endoscopic ultrasonography in determining the depth of invasion and indication for endoscopic treatment of early gastric cancer. J Clin Gastroenterol. 2009;43:318–22. 19077733 10.1097/MCG.0b013e3181775966

[15] Akahoshi K, Chijiwa Y, Hamada S, et al. Pretreatment staging of endoscopically early gastric cancer with a 15 MHz ultrasound catheter probe. Gastrointest Endosc. 1998;48:470–6. 9831834 10.1016/s0016-5107(98)70087-2

[16] Choi J, Kim SG, Im JP, et al. Comparison of endoscopic ultrasonography and conventional endoscopy for prediction of depth of tumor invasion in early gastric cancer. Endoscopy. 2010;42:705–13. 20652857 10.1055/s-0030-1255617

[17] Pollack BJ, Chak A, Sivak MV. Endoscopic ultrasonography. Semin Oncol. 1996;23:336–46. 8658217

[18] LeCun Y, Bengio Y, Hinton G. Deep learning. Nature. 2015;521:436–44. 26017442 10.1038/nature14539

[19] Hirasawa T, Aoyama K, Tanimoto T, et al. Application of artificial intelligence using a convolutional neural network for detecting gastric cancer in endoscopic images. Gastric Cancer. 2018;21:653–60. 29335825 10.1007/s10120-018-0793-2

[20] Wu L, Zhou W, Wan X, et al. A deep neural network improves endoscopic detection of early gastric cancer without blind spots. Endoscopy. 2019;51:522–31. 30861533 10.1055/a-0855-3532

[21] Luo H, Xu G, Li C, et al. Real-time artificial intelligence for detection of upper gastrointestinal cancer by endoscopy: a multicentre, case-control, diagnostic study. Lancet Oncol. 2019;20:1645–54. 31591062 10.1016/S1470-2045(19)30637-0

[22] Li L, Chen Y, Shen Z, et al. Convolutional neural network for the diagnosis of early gastric cancer based on magnifying narrow band imaging. Gastric Cancer. 2020;23:126–32. 31332619 10.1007/s10120-019-00992-2 PMC6942561

[23] Hu H, Gong L, Dong D, et al. Identifying early gastric cancer under magnifying narrow-band images with deep learning: a multicenter study. Gastrointest Endosc. 2021;93:1333–41.e3. 33248070 10.1016/j.gie.2020.11.014

[24] An P, Yang D, Wang J, et al. A deep learning method for delineating early gastric cancer resection margin under chromoendoscopy and white light endoscopy. Gastric Cancer. 2020;23:884–92. 32356118 10.1007/s10120-020-01071-7

[25] Ling T, Wu L, Fu Y, et al. A deep learning-based system for identifying differentiation status and delineating the margins of early gastric cancer in magnifying narrow-band imaging endoscopy. Endoscopy. 2021;53:469–77. 32725617 10.1055/a-1229-0920

[26] Zhu Y, Wang QC, Xu MD, et al. Application of convolutional neural network in the diagnosis of the invasion depth of gastric cancer based on conventional endoscopy. Gastrointest Endosc. 2019;89:806–15.e1. 30452913 10.1016/j.gie.2018.11.011

[27] Nagao S, Tsuji Y, Sakaguchi Y, et al. Highly accurate artificial intelligence systems to predict the invasion depth of gastric cancer: efficacy of conventional white-light imaging, nonmagnifying narrow-band imaging, and indigo-carmine dye contrast imaging. Gastrointest Endosc. 2020;92:866–73.e1. 32592776 10.1016/j.gie.2020.06.047

[28] Hamada K, Kawahara Y, Tanimoto T, et al. Application of convolutional neural networks for evaluating the depth of invasion of early gastric cancer based on endoscopic images. J Gastroenterol Hepatol. 2022;37:352–7. 34713495 10.1111/jgh.15725

[29] Zhu JY, Park T, Isola P, et al. Unpaired image-to-image translation using cycle-consistent adversarial networks. In: Proceedings of the IEEE international conference on computer vision. Venice, Italy: IEEE; 2017. p. 2223–32.

[30] Park CH, Park JC, Kim EH, et al. Learning curve for EUS in gastric cancer T staging by using cumulative sum analysis. Gastrointest Endosc. 2015;81:898–905.e1. 25442086 10.1016/j.gie.2014.08.024

[31] Campanella G, Hanna MG, Geneslaw L, et al. Clinical-grade computational pathology using weakly supervised deep learning on whole slide images. Nat Med. 2019;25:1301–9. 31308507 10.1038/s41591-019-0508-1 PMC7418463

[32] De Fauw J, Ledsam JR, Romera-Paredes B, et al. Clinically applicable deep learning for diagnosis and referral in retinal disease. Nat Med. 2018;24:1342–50. 30104768 10.1038/s41591-018-0107-6

[33] Stacke K, Eilertsen G, Unger J, et al. Measuring domain shift for deep learning in histopathology. IEEE J Biomed Health Inform. 2021;25:325–36. 33085623 10.1109/JBHI.2020.3032060

[34] Yang J, Dvornek NC, Zhang F, et al. Unsupervised domain adaptation via disentangled representations: application to cross-modality liver segmentation. Med Image Comput Comput Assist Interv. 2019;11765:255–63. 32377643 10.1007/978-3-030-32245-8_29 PMC7202929

[35] Park J, Hwang Y, Kim HG, et al. Reduced detection rate of artificial intelligence in images obtained from untrained endoscope models and improvement using domain adaptation algorithm. Front Med (Lausanne). 2022;9:1036974. 36438041 10.3389/fmed.2022.1036974 PMC9684642

[36] Wu L, Wang J, He X, et al. Deep learning system compared with expert endoscopists in predicting early gastric cancer and its invasion depth and differentiation status (with videos). Gastrointest Endosc. 2022;95:92–104. 34245752 10.1016/j.gie.2021.06.033

[37] Goto A, Kubota N, Nishikawa J, et al. Cooperation between artificial intelligence and endoscopists for diagnosing invasion depth of early gastric cancer. Gastric Cancer. 2023;26:116–22. 36040575 10.1007/s10120-022-01330-9 PMC9813068

[38] Gong EJ, Bang CS, Lee JJ, et al. Deep learning-based clinical decision support system for gastric neoplasms in real-time endoscopy: development and validation study. Endoscopy. 2023;55:701–8. 36754065 10.1055/a-2031-0691

[39] Kuroki K, Oka S, Tanaka S, Yorita N, et al. Clinical significance of endoscopic ultrasonography in diagnosing invasion depth of early gastric cancer prior to endoscopic submucosal dissection. Gastric Cancer. 2021;24:145–55. 32572791 10.1007/s10120-020-01100-5

