# Supplementary material for: A novel artificial intelligence-based endoscopic ultrasonography diagnostic system for diagnosing the invasion depth of early gastric cancer
Source: J Gastroenterol. 2024 May 7;59(7):543–55. doi: 10.1007/s00535-024-02102-1 (PMC11217111; doi:10.1007/s00535-024-02102-1)
Supplement: Supplementary file 1 — Supplementary file1 (DOCX 3764 KB) [file 535_2024_2102_MOESM1_ESM.docx]

**Supplementary materials**

**Supplementary method**

***Establishment of a diagnostic algorithm***

With the Development dataset, our AI model was trained using 5-fold cross-validation (Supplementary Fig. 1a). Through training, all hyperparameters were tuned. Our model calculates the quality score, invasion score, and noninvasion score for each EUS image. These scores offer image-level diagnostics, and we explored algorithms to integrate them for lesion-wise diagnosis. By combining the scores in the validation fold, we calculated the overall quality score, invasion score, and noninvasion score for the entire development dataset (Supplementary Fig. 1a). Upon examining the relationships among these three scores, they appeared to exhibit a relationship of mutual exclusion (Supplementary Fig. 1b). While there were few differences in the distribution patterns of the quality score and noninvasion score between 'M-SM1' and 'SM2 or deeper' lesions, the invasion score displayed a pronounced difference in distribution (Supplementary Fig. 1c). Based on the hypothesis that the invasion score plays a significant role in the depth diagnosis, we calculated the diagnostic performance using ROC curves when setting the maximum, average, and median values of the three scores as the score for each lesion (Supplementary Fig. 1d). As a result, the maximum invasion score showed the highest diagnostic accuracy, with an AUC of 0.747. Therefore, in our AI model, the scores were calculated for all EUS images for each lesion, and the highest invasion score among them was treated as the score for that lesion.

**
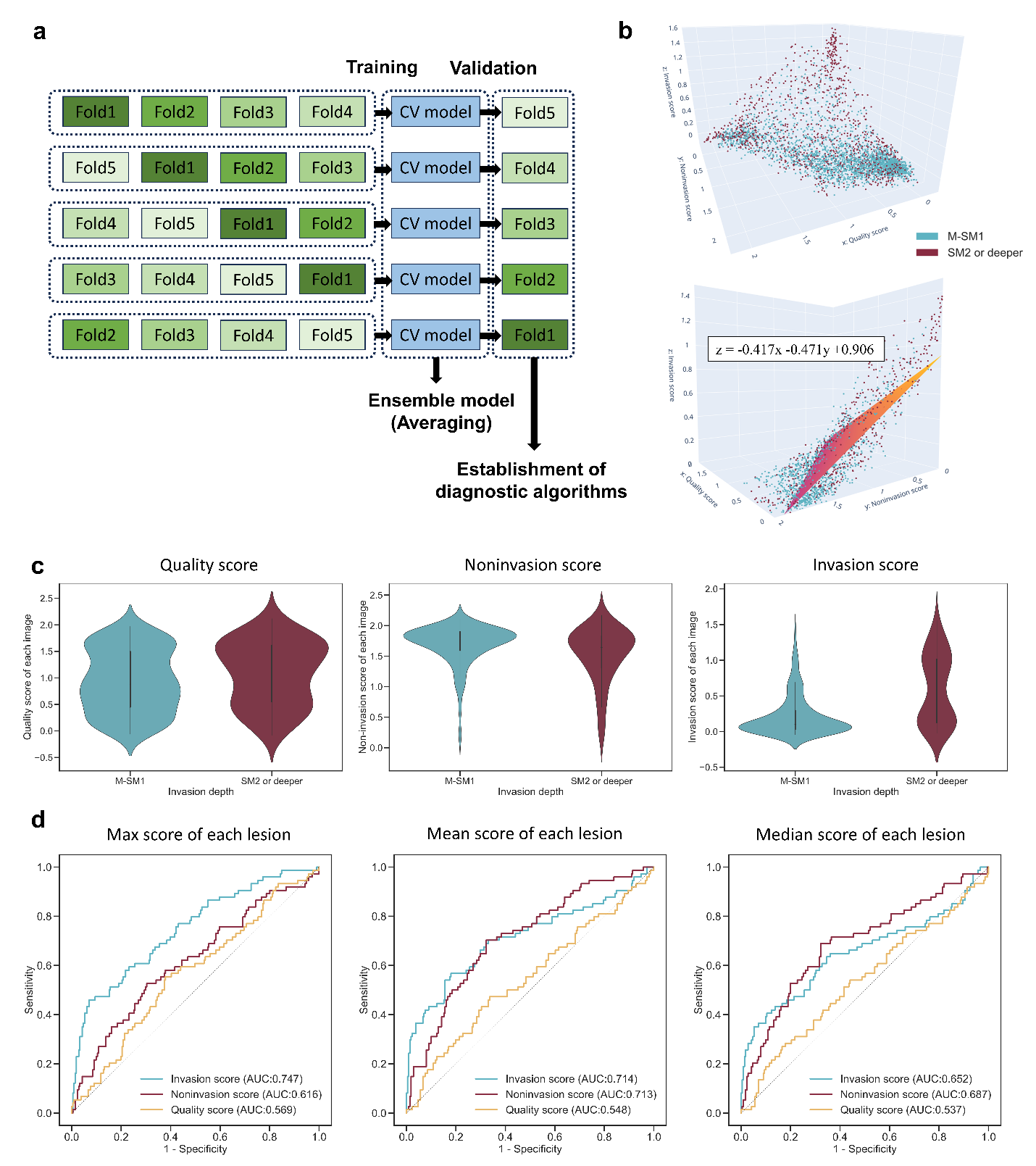
****Supplementary Fig. 1** Establishment of a diagnostic algorithm with the development dataset. **a** 5-fold cross-validation was conducted. We integrated the scores from the validation fold and used them to establish the diagnostic algorithm. **b** Correlations between the quality score (X-axis), noninvasion score (Y-axis), and invasion score (Z-axis) for all images in the development dataset. Plot of the linear regression plane of these points, showing that when one score was high, the other scores were not as high, indicating a correlation between them. **c** Violin plots of each image's score grouped by depth of invasion. There was a noticeable skew in the distribution of the invasion score. **d** The diagnostic performance when setting the maximum, average, and median values of the three scores as the score for each lesion. CV, cross-validation, M-SM1, mucosal cancer or cancer in the submucosa <500 μm from the muscularis mucosae; SM2, cancer in the submucosa ≥500 μm from the muscularis mucosae


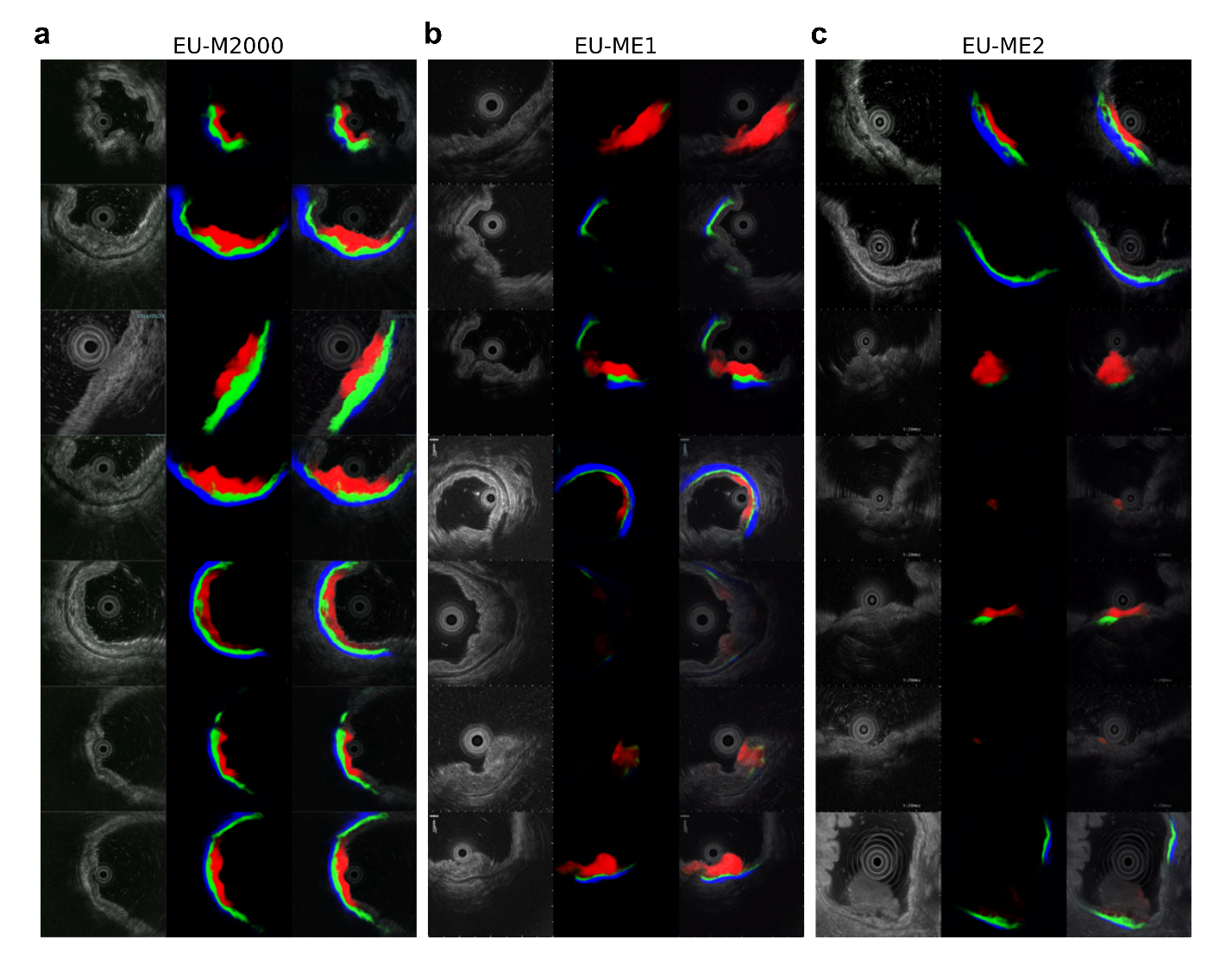
**Supplementary Fig. 2** Segmented images for each EUS system in the external validation dataset. **a** EU-M2000, **b** EU-ME1, **c** EU-ME2. Seven randomly selected images were extracted and presented. From left to right, the original EUS image, the segmented image output from the segmentation model, and the merged image are presented. Good-quality segmented images were obtained from the EU-M2000-derived images, whereas the quality of segmentation was lower for the EU-ME1- and EU-ME2-derived images. EU-M2000-derived images were included in the development dataset, but EU-ME1- and EU-ME2-derived images were not, suggesting the presence of a domain shift problem
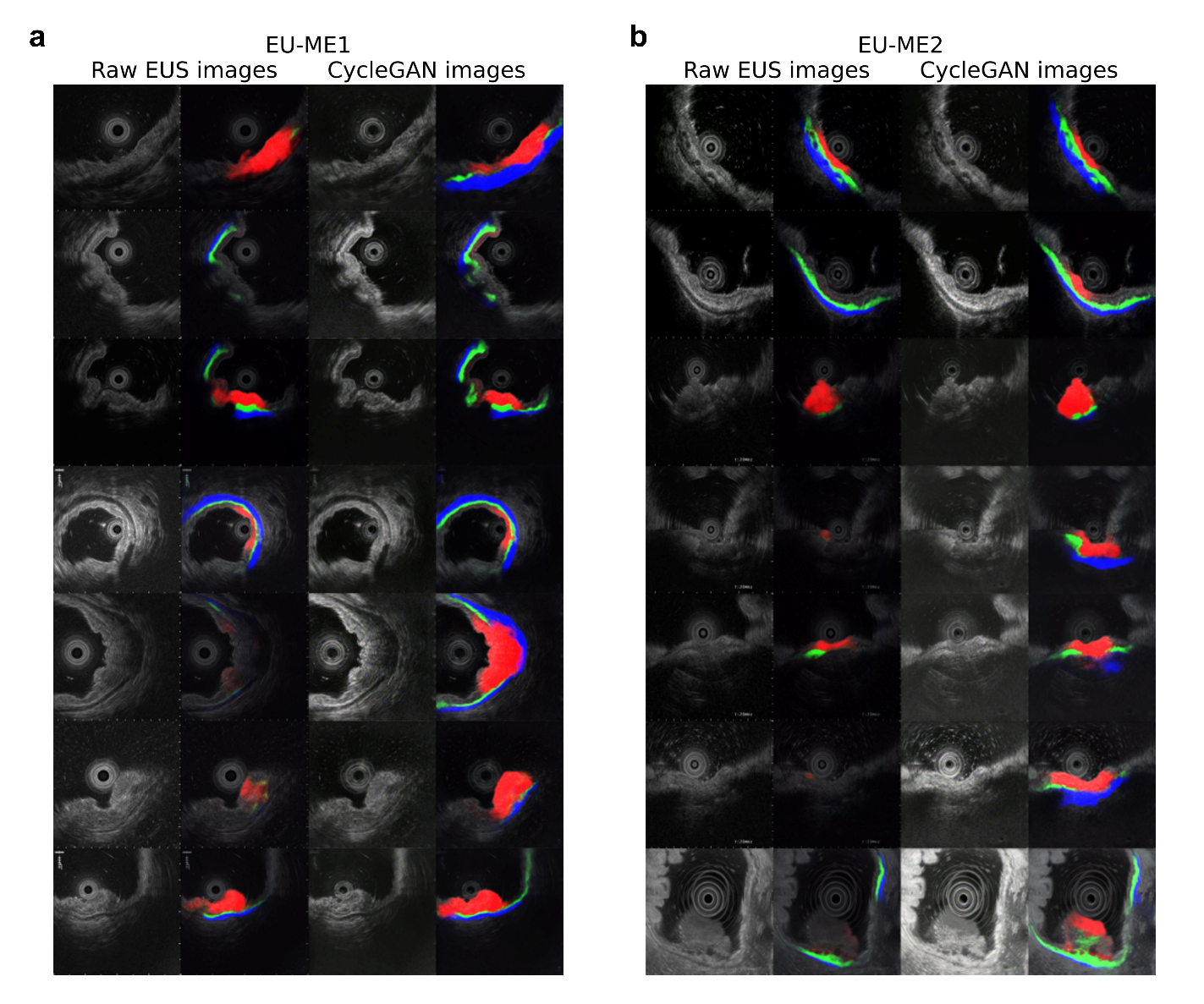


**Supplementary Fig. 3** Improvement of segmentation quality using domain adaptation with CycleGAN. CycleGAN was applied to the selected EUS images in Supplementary Fig. 2, and the output of the segmentation model is shown. **a** EU-ME1, **b** EU-ME2. Most images showed improved segmentation quality

**Supplementary Table 1.** Hyperparameters of the first step segmentation model

| **Hyperparameter** | **Value** |
| --- | --- |
| Dataset information |  |
| Training images | 454 |
| Validation images | 43 |
| Model hyperparameters |  |
| Base model (Encoder) | U-NET (ResNet-34) |
| Input image size | 512 x 512 pixels |
| No. of epochs | 500 |
| Early stopping | Yes |
| Batch size | 4 |
| Loss function | Binary cross-entropy with logits loss |
| Optimization algorithm | Adam (learning rate: 0.0005) |
| Training indicator | Dice coefficient |
| Learning rate scheduler | ReduceLROnPlateau method (factor=0.1, patience=10) |
| Augmentation hyperparameters |  |
| Flipping (frequency) | Horizontal (*p=0.5*) |
| Scaling (frequency) | Range 0.9 to 1.1 (*p=0.9*) |
| Rotation (frequency) | Range -180°to 180° (*p=0.9*) |
| Brightness shift (frequency) | Range -0.5 to 0.5 (*p=0.9*) |
| Contrast shift (frequency) | Range -0.5 to 0.5 (*p=0.9*) |
| Normalization | Mean [0.485, 0.456, 0.406]  Standard deviation [0.229, 0.224, 0.225] |

**Supplementary Table 2.** Hyperparameters of the second step classification model

| **Hyperparameter** | **Value** |
| --- | --- |
| Dataset information |  |
| Fold 1 | Train 2726 (228 patients), Validation 725 (57 patients) |
| Fold 2 | Train 2763 (228 patients), Validation 688 (57 patients) |
| Fold 3 | Train 2724 (228 patients), Validation 727 (57 patients) |
| Fold 4 | Train 2855 (228 patients), Validation 596 (57 patients) |
| Fold 5 | Train 2736 (228 patients), Validation 715 (57 patients) |
| Model hyperparameters |  |
| Base model (Pretrained weight) | EfficientnetV2L (ImageNet-1K) |
| Input image size | 224 x 224 pixels |
| Mix-up ratio of input images | Raw image : mask image (generated by segmentation model) = 1.0 : 0.2 |
| No. of epochs | 50 |
| Early stopping | Yes |
| Batch size | 8 |
| Loss function | Root Mean Squared Error |
| Optimization algorithm | RAdam (learning rate: 0.0001) |
| Training indicator | Mean AUROC of the whole score (Quality score, Noninvasion score, Invasion score) |
| Augmentation hyperparameters |  |
| Flipping (frequency) | Horizontal (*p=0.5*) |
| Scaling (frequency) | Range 0.9 to 1.1 (*p=0.9*) |
| Rotation (frequency) | Range -45°to 45°(*p=0.9*) |
| Brightness shift (frequency) | Range -0.5 to 0.5 (*p=0.9*) |
| Contrast shift (frequency) | Range -0.5 to 0.5 (*p=0.9*) |
| Normalization | Mean [0.485, 0.456, 0.406]  Standard deviation [0.229, 0.224, 0.225] |

| **Characteristics** | **Development**  **(n=285)** | **Internal validation**  **(n=135)** | **External validation**  **(n=139)** |
| --- | --- | --- | --- |
| Study period | Apr 2009 to Dec 2010  Jan 2013 to Dec 2018 | Jan 2011 to Dec 2012  Jan 2019 to Dec 2019 | May 2017 to Jan 2021 |
| Age, years, median (range) | 71 (30-89) | 73 (34-88) | 73 (39-87) |
| Sex, n (%) |  |  |  |
| Male | 214 (75) | 102 (76) | 101 (73) |
| Female | 71 (25) | 33 (24) | 38 (27) |
| Tumor size, mm, median (range) | 20 (3-100) | 18 (2-90) | 24 (2-70) |
| Lesion location, n (%) |  |  |  |
| Upper | 59 (21) | 26 (19) | 30 (21) |
| Middle | 93 (33) | 50 (37) | 54 (39) |
| Lower | 133 (47) | 59 (44) | 55 (40) |
| Macroscopic type, n (%) |  |  |  |
| Elevated | 17 (6) | 4 (3) | 9 (6) |
| Flat | 251 (88) | 117 (87) | 112 (81) |
| Depressed | 0 (0) | 2 (1) | 4 (3) |
| Mixed | 17 (6) | 12 (9) | 14 (10) |
| Histological depth, n (%) |  |  |  |
| M | 182 (64) | 86 (64) | 58 (42) |
| SM1 | 34 (12) | 11 (8) | 14 (10) |
| SM2 or deeper | 69 (24) | 38 (28) | 67 (48) |
| Histological type, n (%) |  |  |  |
| Differentiated | 240 (84) | 108 (80) | 87 (63) |
| Mixed | 25 (9) | 11 (8) | 32 (23) |
| Undifferentiated | 19 (7) | 13 (10) | 14 (10) |
| Other than above | 1 (0) | 3 (2) | 6 (4) |
| EUS equipment, n (%) |  |  |  |
| EU-M2000 (Olympus) | 229 (80) | 109 (81) | 4 (3) |
| EU-ME1 (Olympus) | 0 (0) | 0 (0) | 25 (18) |
| EU-ME2 (Olympus) | 0 (0) | 0 (0) | 109 (78) |
| SP-702 (Fujifilm) | 0 (0) | 0 (0) | 1 (1) |
| SP-900 (Fujifilm) | 56 (20) | 26 (19) | 0 (0) |
| Treatment, n (%) |  |  |  |
| ESD | 192 (67) | 88 (65) | 77 (55) |
| Surgery | 93 (33) | 47 (35) | 62 (45) |

**Supplementary Table 3.** Characteristics of the patients

M, mucosal cancer; SM1, cancer in the submucosa less than 500 μm from the muscularis mucosae; SM2, cancer in the submucosa greater than or equal to 500 μm from the muscularis mucosae; ESD endoscopic submucosal dissection.

**Supplementary Table 4.** Thresholds of invasion score and the diagnostic performance

| **Threshold of invasion score** | **Number of SM2 or deeper EGC above threshold** | **Accuracy** | **Sensitivity** | **Specificity** | **PPV** | **NPV** | **F1 score** |
| --- | --- | --- | --- | --- | --- | --- | --- |
| 1.394 | 1 | 0.704 | 0.024 | 1.000 | 1.000 | 0.701 | 0.048 |
| 0.693 | 14 | 0.800 | 0.341 | 1.000 | 1.000 | 0.777 | 0.509 |
| 0.644 | 14 | 0.793 | 0.341 | 0.989 | 0.933 | 0.775 | 0.500 |
| 0.630 | 15 | 0.800 | 0.366 | 0.989 | 0.938 | 0.782 | 0.526 |
| 0.566 | 15 | 0.793 | 0.366 | 0.979 | 0.882 | 0.780 | 0.517 |
| 0.548 | 16 | 0.800 | 0.390 | 0.979 | 0.889 | 0.786 | 0.542 |
| 0.490 | 16 | 0.793 | 0.390 | 0.968 | 0.842 | 0.784 | 0.533 |
| 0.428 | 21 | 0.830 | 0.512 | 0.968 | 0.875 | 0.820 | 0.646 |
| 0.409 | 21 | 0.807 | 0.512 | 0.936 | 0.778 | 0.815 | 0.618 |
| 0.406 | 22 | 0.815 | 0.537 | 0.936 | 0.786 | 0.822 | 0.638 |
| 0.403 | 22 | 0.807 | 0.537 | 0.926 | 0.759 | 0.821 | 0.629 |
| 0.361 | 24 | 0.822 | 0.585 | 0.926 | 0.774 | 0.837 | 0.667 |
| 0.334 | 24 | 0.807 | 0.585 | 0.904 | 0.727 | 0.833 | 0.649 |
| **0.306*** | **26** | **0.822** | **0.634** | **0.904** | **0.743** | **0.850** | **0.684** |
| 0.271 | 26 | 0.793 | 0.634 | 0.862 | 0.667 | 0.844 | 0.650 |
| 0.254 | 30 | 0.822 | 0.732 | 0.862 | 0.698 | 0.880 | 0.714 |
| 0.252 | 30 | 0.807 | 0.732 | 0.840 | 0.667 | 0.878 | 0.698 |
| 0.237 | 31 | 0.815 | 0.756 | 0.840 | 0.674 | 0.888 | 0.713 |
| 0.198 | 31 | 0.763 | 0.756 | 0.766 | 0.585 | 0.878 | 0.660 |
| 0.190 | 32 | 0.770 | 0.780 | 0.766 | 0.593 | 0.889 | 0.674 |
| 0.160 | 32 | 0.719 | 0.780 | 0.691 | 0.525 | 0.878 | 0.627 |
| 0.159 | 33 | 0.726 | 0.805 | 0.691 | 0.532 | 0.890 | 0.641 |
| 0.156 | 33 | 0.711 | 0.805 | 0.670 | 0.516 | 0.887 | 0.629 |
| 0.155 | 34 | 0.719 | 0.829 | 0.670 | 0.523 | 0.900 | 0.642 |
| 0.145 | 34 | 0.711 | 0.829 | 0.660 | 0.515 | 0.899 | 0.636 |
| 0.139 | 35 | 0.719 | 0.854 | 0.660 | 0.522 | 0.912 | 0.648 |
| 0.137 | 35 | 0.711 | 0.854 | 0.649 | 0.515 | 0.910 | 0.642 |
| 0.132 | 37 | 0.726 | 0.902 | 0.649 | 0.529 | 0.938 | 0.667 |
| 0.124 | 37 | 0.711 | 0.902 | 0.628 | 0.514 | 0.937 | 0.655 |
| 0.122 | 38 | 0.719 | 0.927 | 0.628 | 0.521 | 0.952 | 0.667 |
| 0.120 | 38 | 0.711 | 0.927 | 0.617 | 0.514 | 0.951 | 0.661 |
| 0.120 | 39 | 0.719 | 0.951 | 0.617 | 0.520 | 0.967 | 0.672 |
| 0.058 | 39 | 0.585 | 0.951 | 0.426 | 0.419 | 0.952 | 0.582 |
| 0.055 | 40 | 0.593 | 0.976 | 0.426 | 0.426 | 0.976 | 0.593 |
| 0.022 | 40 | 0.459 | 0.976 | 0.234 | 0.357 | 0.957 | 0.523 |
| 0.021 | 41 | 0.467 | 1.000 | 0.234 | 0.363 | 1.000 | 0.532 |

SM2, cancer in the submucosa greater than or equal to 500 μm from the muscularis mucosae; EGC, early gastric cancer; PPV, positive predictive value; NPV, negative predictive value.

* The threshold of the invasion score employed in this study.

**Supplementary Table 5.** EUS equipment, number of lesions, image size, and crop size at each institution

| **Dataset** | **Institution** | **Number of lesions** | **EUS equipment** | **Number of patients per equipment** | **Stored images size** | **cropped EUS area size** |
| --- | --- | --- | --- | --- | --- | --- |
| Development and internal validation | Osaka University Hospital | 420 | EU-M2000 (Olympus) | 338 | 640×480 pixels | 388×388 pixels |
|  |  |  | SP-900 (Fujifilm) | 82 | 640×480 pixels | 314×307 pixels |
| External validation | Osaka International Cancer Institute | 47 | EU-ME1 (Olympus) | 19 | 640×480 pixels | 386×389 pixels |
|  |  |  | EU-ME2 (Olympus) | 28 | 1440×1080 pixels | 831×730 pixels |
|  | Kansai Rosai Hospital | 41 | EU-ME2 (Olympus) | 40 | 1440×1080 pixels | 1109×974 pixels |
|  |  |  | SP-702 (Fujifilm) | 1 | 640×480 pixels | 462×437 pixels |
|  | Toyonaka Municipal Hospital | 19 | EU-M2000 (Olympus) | 3 | 640×480 pixels | 433×366 pixels |
|  |  |  | EU-ME2 (Olympus) | 16 | 1440×1080 pixels | 870×865 pixels |
|  | Osaka General Medical Center | 10 | EU-ME2 (Olympus) | 10 | 1440×1080 pixels | 832×730 pixels |
|  | Suita Municipal Hospital | 7 | EU-ME2 (Olympus) | 7 | 1024×576 pixels, 1024×768 pixels | 592×518 pixels, 717×715 pixels, 790×693 pixels |
|  | Itami City Hospital | 5 | EU-ME1 (Olympus) | 5 | 640×480 pixels, 1169×877 pixels | 395×392 pixels, 721×713 pixels, |
|  | Osaka Rosai Hospital | 4 | EU-M2000 (Olympus) | 1 | 640×480 pixels | 381×387 pixels |
|  |  |  | EU-ME2 (Olympus) | 3 | 1024×576 pixels | 593×519 pixels |
|  | Ikeda Municipal Hospital | 3 | EU-ME2 (Olympus) | 3 | 1676×1080 pixels | 1112×973 pixels |
|  | Sumitomo Hospital | 2 | EU-ME1 (Olympus) | 1 | 640×480 pixels | 486×407 pixels |
|  |  |  | EU-ME2 (Olympus) | 1 | 640×480 pixels | 423×369 pixels |
|  | Yao Municipal Hospital | 1 | EU-ME2 (Olympus) | 1 | 640×480 pixels | 415×369 pixels |
